# SGLT2 inhibition attenuates arterial dysfunction and decreases vascular F-actin content and expression of proteins associated with oxidative stress in aged mice

**DOI:** 10.1007/s11357-022-00563-x

**Published:** 2022-04-15

**Authors:** Rogerio N. Soares, Francisco I. Ramirez-Perez, Francisco J. Cabral-Amador, Mariana Morales-Quinones, Christopher A. Foote, Thaysa Ghiarone, Neekun Sharma, Gavin Power, James A. Smith, R. Scott Rector, Luis A. Martinez-Lemus, Jaume Padilla, Camila Manrique-Acevedo

**Affiliations:** 1 Department of Medicine, University of Missouri, Columbia, MO USA; 2 Department of Medical Pharmacology and Physiology, University of Missouri, Columbia, MO USA; 3 Department of Nutrition and Exercise Physiology, University of Missouri, Columbia, MO USA; 4 Research Service, Harry S. Truman Memorial Veterans’ Hospital, Columbia, MO USA; 5 Division of Gastroenterology and Hepatology, Department of Medicine, University of Missouri, Columbia, MO USA; 6 Dalton Cardiovascular Research Center, University of Missouri, Columbia, MO USA; 7 Department of Biomedical, Biological and Chemical Engineering, University of Missouri, Columbia, MO USA; 8 Division of Endocrinology, Diabetes and Metabolism, Department of Medicine, University of Missouri, Columbia, MO USA

**Keywords:** SGLT2, Arterial stiffness, Aging, Endothelial function, Oxidative stress

## Abstract

Aging of the vasculature is characterized by endothelial dysfunction and arterial stiffening, two key events in the pathogenesis of cardiovascular disease (CVD). Treatment with sodium glucose transporter 2 (SGLT2) inhibitors is now known to decrease cardiovascular morbidity and mortality in type 2 diabetes. However, whether SGLT2 inhibition attenuates vascular aging is unknown. We first confirmed in a cohort of adult subjects that aging is associated with impaired endothelial function and increased arterial stiffness and that these two variables are inversely correlated. Next, we investigated whether SGLT2 inhibition with empagliflozin (Empa) ameliorates endothelial dysfunction and reduces arterial stiffness in aged mice with confirmed vascular dysfunction. Specifically, we assessed mesenteric artery endothelial function and stiffness (via flow-mediated dilation and pressure myography mechanical responses, respectively) and aortic stiffness (in vivo via pulse wave velocity and ex vivo via atomic force microscopy) in Empa-treated (14 mg/kg/day for 6 weeks) and control 80-week-old C57BL/6 J male mice. We report that Empa-treated mice exhibited improved mesenteric endothelial function compared with control, in parallel with reduced mesenteric artery and aortic stiffness. Additionally, Empa-treated mice had greater vascular endothelial nitric oxide synthase activation, lower phosphorylated cofilin, and filamentous actin content, with downregulation of pathways involved in production of reactive oxygen species. Our findings demonstrate that Empa improves endothelial function and reduces arterial stiffness in a preclinical model of aging, making SGLT2 inhibition a potential therapeutic alternative to reduce the progression of CVD in older individuals.

**Graphical abstract:**

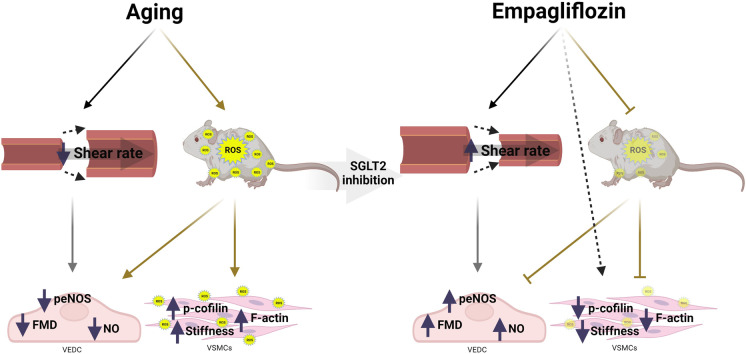

**Supplementary Information:**

The online version contains supplementary material available at 10.1007/s11357-022-00563-x.

## Introduction

Cardiovascular disease (CVD) is the main cause of death in older adults in the USA [1, 2]. The burden of CVD associated with aging is expected to grow in parallel with a steady increase in the percentage of older population [1]. Endothelial dysfunction and arterial stiffening are key occurrences in the genesis and progression of hypertension, chronic kidney disease, and heart failure in older adults [3–6]. Further, aging-related arterial dysfunction and stiffening are accompanied by an increase in arterial internal diameter and wall thickness [7], vascular adaptations that further compromise vascular function.

Therapeutic strategies such as lifestyle modifications (weight loss and increased physical activity), antihypertensive therapy, and lipid-lowering medications have shown variable effectiveness at improving endothelial function and ameliorating arterial stiffening and remodeling in older adults [2, 6, 8–10]. Thus, additional therapeutic approaches aimed at improving vascular health in older individuals are needed. In this regard, evidence from different clinical trials demonstrates that inhibition of the sodium glucose co-transporter 2 (SGLT2) results in decreased cardiovascular events and CVD-related mortality in both patients with and without diabetes [11–13]. SGLT2 co-transporters are predominantly located in the proximal renal tubules and are responsible for reabsorption of 90% of the glucose in the glomerular filtrate [14]. Indeed, while SGLT2 inhibitors were originally designed as glucose lowering agents [15], growing evidence supports their beneficial, non-glucose lowering dependent, renal, and cardiovascular effects. In particular, the SGLT2 inhibitor empagliflozin (Empa) has been shown to reduce cardiovascular mortality and nonfatal myocardial infarction, stroke, and cardiovascular death regardless of the presence of type 2 diabetes [13, 16–18]. Notwithstanding, the well-documented therapeutic effects of SGLT2 inhibitors, potential side effects of these agents such as genitourinary infections, volume depletion, and increased risk of diabetes ketoacidosis have also been recognized [19].

Mechanisms postulated to explain the beneficial cardiovascular properties of SGLT2 inhibition include weight loss and antihypertensive effect, diuresis-induced blood volume reduction, increased red blood cell mass, improved myocardial bioenergetics, decreased arterial stiffness, and improved endothelial function [20–25]. However, the extent to which the favorable cardiovascular effects of SGLT2 inhibitors are translatable to aging remains unknown. Given the above, the potential impact of SGLT2 inhibition on aging-related endothelial dysfunction, arterial stiffening, and remodeling warrants investigation.

Herein, we first confirmed in a cohort of adult human subjects that aging is associated with impaired endothelial function and increased arterial stiffness and that these two variables are inversely correlated. Next, we investigated whether treatment with the SGLT2 inhibitor, Empa, for 6 weeks ameliorates endothelial dysfunction and reduces arterial stiffness in aged mice with confirmed vascular dysfunction. Complementary experiments were also performed in cultured vascular smooth muscle cells treated with Empa.

## Methods

### Human subjects

All experimental procedures performed in human subjects were approved by the University of Missouri Institutional Review Board (protocols #2,012,990, #2,028,142 and #2,012,869) and conducted following the *Declaration of Helsinki*. Written informed consent was obtained from all subjects prior to participation. Exclusion criteria included nicotine use, pregnancy, and excessive alcohol consumption (> 14 drinks per week for men and > 7 drinks per week for women). Eighteen (9 M/9 F) healthy young (25 ± 1 years of age) and eighteen (9 M/9 F) older adult participants (61 ± 1 years of age) were admitted to the Clinical Research Center at the University of Missouri Hospital after an overnight fast.

After body weight and height measurements were obtained, participants rested supine for 15 min prior to the vascular assessments as previously described [26]. Arterial stiffness was measured by assessing carotid-to-femoral pulse wave velocity (cfPWV) using a cuff-based SphygmoCor XCEL (AtCor Medical, Itasca, IL). The SphygmoCor XCEL device allows simultaneous acquisition of carotid (via tonometer) and femoral (via cuff) pulse waves. Transit time between carotid and femoral pressure waves was calculated with the foot-to-foot method. Popliteal artery flow-mediated dilation (FMD) was assessed as previously described [26, 27]. Popliteal artery diameter and blood velocity were measured using high-resolution duplex-Doppler ultrasound according to current guidelines [28]. Recordings of all vascular variables were analyzed off-line by a blinded investigator using specialized edge detection software (Cardiovascular Suite; Quipu, Pisa, Italy). The %FMD was calculated as the change in peak diameter during the reperfusion phase divided by baseline diameter times 100.

### Animals

All animal procedures were performed in accordance with the Animal Use and Care Committee at the University of Missouri in Columbia. The University of Missouri is accredited by the AAALAC International. For an initial vascular characterization of the model, young (12 weeks old, *n* = 5) and old (86 weeks old, *n* = 5) C57BL/6 J male mice were obtained from The Jackson Laboratory (Bar Harbor, ME). In order to investigate the effects of Empa on vascular aging, 72-week-old male mice, also obtained from the The Jackson Laboratory, were divided into two groups and fed for 6 weeks with (1) Empa-enriched diet (*n* = 29; LabDiet 5053 w/ 133 ppm Empa; Test Diet®, Richmond, IN) with a calculated dose of 14 mg/kg/day or (2) standard chow (*n* = 29; LabDiet 5053). Young mice were not treated with Empa because these mice do not exhibit arterial dysfunction. Different cohorts of mice were used for the experimental procedures described below. All mice were provided water ad libitum while cohoused in an environmentally controlled facility maintained at 24 °C on a 12-h light:dark cycle from 0700 to 1900 h. Two mice (control, *n* = 1; and Empa, *n* = 1) died during the intervention. All procedures were performed by investigators that were blinded to the experimental conditions.

### Tail-cuff blood pressure assessment

Following acclimatization, blood pressure was determined noninvasively using a CODA tail-cuff blood pressure system (CODA-HT2; Kent Scientific, Torrington, CT) at 5 weeks after the beginning of the Empa or control intervention. A minimum of eight blood pressure readings were recorded and averaged for each animal.

### Glucose tolerance test

Assessment of glucose tolerance was performed as previously described [29]. After a 4-h fast, the tail vein was nicked with a scalpel, and blood sampled with a hand-held glucometer (AlphaTrak 2, Abbott Laboratories, Abbott Park, IL). A baseline measure of glucose was determined before administering a sterile solution of 50% dextrose (1.0 g/kg body weight) via intraperitoneal injection. Glucose levels were measured after 15, 30, 45, 60, and 120 min. Glucose area under curve (AUC) was calculated using the trapezoidal rule.

### *Doppler ultrasound-derived *in vivo* measurements of thoracic aorta internal diameter and blood flow*

One week prior to sacrifice, thoracic aorta internal diameter and blood flow were determined via Doppler ultrasound (Vevo 2100, FUJIFILM VisualSonics Inc., Toronto, Canada) and recorded for offline analysis using a wall-tracking software program (Cardiovascular Suite version 4.2.1, Quipu srl, Pisa, Italy). Briefly, mice were anesthetized with 1.75% isoflurane and placed in supine position on a heated platform (42 °C). Hair was removed from the thoracic region. Heart rate was continuously monitored via ECG. Using a high-frequency ultrasound probe (MS700), the thoracic aorta was identified, and vessel internal diameter and mean blood velocity were determined as the average of six measurements over a 2-min period, each measurement resulting from the average velocity across five consecutive cardiac cycles.

### *In vivo and *ex vivo* assessment of aortic stiffness*

In vivo and ex vivo evaluations of aortic stiffness were performed as previously described [30]. Briefly, 2 days prior to sacrifice, in vivo aortic stiffness was evaluated in isoflurane-anesthetized mice (1.75% in 100% oxygen stream) by pulse-wave velocity (PWV) using Doppler ultrasound (Vevo 2100, FUJIFILM VisualSonics Inc.). PWV was determined as the difference in arrival times of a Doppler pulse wave at two locations along the aorta at a known distance and expressed in m/s. Ex vivo aortic stiffness was assessed by a nano-indentation protocol utilizing atomic force microscopy (AFM) and calculated as the force exerted by the AFM probe on the luminal surface of aortic explants as previously described [31]. Briefly, a 2-mm ring of the thoracic aorta was isolated from each mouse following the experimental period. The aortic ring was opened longitudinally, and the adventitial surface of each explant was fastened to a glass cover slip using Cell-Tak to achieve *en face* access to the endothelial surface for placement of the AFM probe. Stiffness of the endothelial surface was estimated by placing the probe at ~ 15 random locations along the luminal surface of the explant and determining the average endothelial stiffness for that aorta.

### Ex vivo* mesenteric artery endothelium-dependent and independent vasodilation*

Mesenteric endothelium-dependent vasodilatory function was assessed via FMD as previously described [32]. Mesenteric artery segments (2nd order) were cannulated, pressurized at 70 mmHg, and warmed to 37 °C in myography chambers (Living Systems Instrumentation, Burlington, VT, USA). Mesenteric artery viability was tested by exposure to 80 mM potassium chloride (KCl). Afterward, mesenteric arteries were pre constricted with 10^−5^ M phenylephrine (Phe) and then exposed to incremental increases in intraluminal flow (0–3 ml/hr). Intraluminal flow was maintained for 3 min at each flow rate with a syringe pump while preserving mean intravascular pressure at 70 mmHg with a pressure-servo system. FMD was calculated as percent and absolute change in mesenteric artery internal diameter in response to consecutive increases in shear stress. Shear stress was calculated using the formula: $\tau =32\frac{\mu Q}{\pi {d}^{3}},$ where $\mu$ is the viscosity of the buffer, $Q$ is the flow rate, and $d$ is vessel internal diameter. Arteries were also exposed to cumulative concentrations of sodium nitroprusside (SNP; 10^−8^ to 10^−4^ M) to evaluate endothelium-independent vasodilatory responses.

### Ex vivo* mesenteric structural remodeling and stiffness*

Following the functional protocol, mesenteric artery stiffness was assessed as previously described [30, 33]. Elastic properties were characterized under passive conditions. Arteries were exposed to step-wise increments in intraluminal pressure. Luminal diameter (*D*) and wall thicknesses (*τ*) were recorded, and mechanical properties were calculated as previously described [30, 33]. The incremental modulus of elasticity (*E*_inc_), *D* and *τ* were measured for a given intraluminal pressure and a density of the intraluminal buffer of *ρ* ≈ 1005 kg/m^3^.

### Histology

A 2-mm segment of the abdominal aorta from the experimental mice was fixed in 4% paraformaldehyde for 48 h. After fixation, tissues were processed for paraffin embedding by a commercial laboratory (IDEXX, Columbia, MO). Five-micrometer serial sections of the aorta were prepared by sectioning throughout the tissue. A section of the abdominal aorta was then subjected to Verhoeff-Von Gieson (VVG) and Masson trichrome stains to measure elastin and collagen content, respectively. The intensity of blue staining (as indicator of collagen content on the Masson trichrome-stained slides) was evaluated from 10 × images and quantified using the Fiji version of Image J following the software directions [34]. Similarly, the content of blue-black staining (as indicator of elastic fibers in VVG-stained slides) was evaluated and quantified using the same software as described above.

### 2-Thiobarbituric acid reactive substance assay

As a marker of systemic oxidative stress, malondialdehyde (MDA) was assessed in plasma from Empa-treated and control mice using a colorimetric/fluorometric assay kit (TBARS assay kit; Cayman Chemical, Ann Arbor, MI) according to the manufacturer’s instructions. The fluorescence from each sample was measured at an excitation wavelength of 530 nm and an emission wavelength of 550 nm in a Biotek plate reader.

### Confocal microscopy

Fixed mesenteric arteries were used to determine the content of filamentous actin (F-actin) stress fibers, phosphorylated cofilin (P-cofilin), nuclei, elastin, collagen, and endothelial nitric oxide (eNOS) and phosphorylated eNOS (P-eNOS). Briefly, vessels were rinsed twice in phosphate-buffered saline (PBS) and once in 0.1 M glycine (5 min each), flushed with PBS, permeabilized with 0.5% Triton X-100 for 15 min followed by washing with PBS, blocked with 1% bovine serum albumin and incubated overnight at 4 °C in primary antibodies vs. P-cofilin (1:50, Cell Signaling, catalogue #3313, Davers, MA), eNOS (1:500, BD Biosciences, catalogue #610,296, Franklin Lakes, NJ), and P-eNOS Ser1177 (1:500, Cell Signaling, catalogue #9571). Vessels were subsequently incubated in secondary antibodies Goat vs. Rabbit Alexa Fluor 488 (Thermo Fisher, catalog #A-11070, Walthan, MA) and Goat vs. Mouse Alexa Fluor 546 (Thermo Fisher, catalog #A-21052), phalloidin-Alexa 633 (Thermo Fisher, catalog #A22283), 4′,6-diamidino-2-phenylindole (DAPI, Sigma-Aldrich catalog #D9542, St Louis, MO), and Alexa Fluor 633 Hydrazide (Molecular Probes, Eugene, OR). Images of nuclei, F-actin, P-cofilin, eNOS, P-eNOS, elastin, and collagen were obtained using a Leica SP5 confocal/multiphoton microscope with a 63 × water immersion 1.2 numerical aperture objective and the following parameters: excitation/emission wavelengths of 840/435–480 nm in multiphoton mode for DAPI, 488/500–550 nm for P-eNOS, 633/645–700 nm for total-cofilin, 543/555–620 nm for F-actin, and 840/400–430 nm for collagen in multiphoton mode capturing second harmonic images. Z-stack images were taken at steps of 0.5 µm from outside the vessel wall to mid-diameter. Imaris software (Bitplane Inc., Concord, MA) was used to render three-dimensional reconstruction images of the vessels and to quantify the volume and intensities of the molecules of interest. Volumetric and intensity data were normalized by the number of smooth muscle cells, when measurements corresponded to the vascular medial layer.

### Cell culture experiments

Confocal microscopy images of human coronary artery vascular smooth muscle cells (VSMC, Thermo Fisher catalogue #C0175C) were obtained from cells plated in µ-angiogenesis 15-well ibidi plates. P-cofilin and F-actin were assessed in cells incubated for 24 h at 37 °C in the absence or presence of Empa 500 nM (Ambeed, catalogue #864,070–44-0, Arlington Heights, IL). At the end of the experiment, cells were fixed in 4% paraformaldehyde, at room temperature for 30 min and permeabilized with 0.5% Triton X-100 for 60 min. Cells were incubated for 24 h with 1:50 P-cofilin (Ser3) (77G2) Rabbit mAb (Cell Signaling, catalogue #3313). This was followed by incubation for 1 h in 1:100 Goat anti-Rabbit IgG secondary antibody Alexa Fluor 635 (Thermo Fisher, catalogue #A-31577), 1:50 Alexa Fluor 568 phalloidin (Thermo Fisher, catalogue #A12380), and 1:500 DAPI to stain P-cofilin, F-actin and nuclei, respectively. Images were acquired with a Leica SPE confocal microscope (Leica Microsystems, Inc., Morrisville, NC). Intensity data were quantified using Imaris software and normalized by the total number of cells.

### Proteomics

Proteomic analysis of the thoracic aortas was performed at Charles W Gehrke Proteomics Center/Research Core Facilities, University of Missouri, as following: Each thoracic aorta sample was homogenized in 0.45 mL of SDS buffer (0.175 M Tris–HCl, pH 8.8, 5% SDS, 15% glycerol, 0.3 M DTT) in the original 1.5-ml tube using a Fisher Scientific 150 Homogenizer with a 5-mm ID stainless steel probe for 2 × 10 s bursts. Samples were then heated at 65 °C for 20 min, and centrifuged for 10 min at 16,000 × g. The supernatant was transferred to a new tube, and four volumes of cold acetone were added to each sample, and protein was precipitated overnight at − 20 °C. Each protein sample was centrifuged and washed with 80% acetone twice, and protein pellets were then resuspended with 6 M urea, 2 M thiourea, and 100 mM ammonium bicarbonate. Protein was quantified using Pierce 660 nm Protein Assay method following the microplate measure instructions in the manual. Details on spectral library built-up, DIA data, statistical analysis, and full list of proteins that showed to be significantly changed with Empa is described on the supplementary material.

### Aortic mitochondrial respiration

Vascular mitochondrial respiration was assessed in isolated thoracic aortae using high-resolution respirometry (Oroboros Oxygraph-2 k; Oroboros Instruments; Innsbruck, Austria). Respiration was measured via oxygen consumption at basal (vessel) and in response to the addition of various substrates to the Oroboros as described previously. [35, 36] Briefly, the thoracic aorta was cleaned and incubated in ice cold BIOPS (10 mM Ca-EGTA buffer, 0.1 μM free calcium, 20 mM imidazole, 20 mM taurine, 50 mM MES, 0.5 mM dithiothreitol, 6.56 mM MgCl2, 5.77 mM ATP, 15 mM phosphocreatine, pH 7.1) for 30 min followed by 40 min in a BIOPS-saponin solution (50 ug/mL). After the saponin incubation, vessels were rinsed twice with MiR05 respiration media (sucrose, 100 mM; K-lactobionate, 60 mM; EGTA, 0.5 mM; MgCl2, 3 mM; taurine, 20 mM; KH2PO4, 10 mM; HEPES, 20 mM; adjusted to pH 7.1 with KOH at 37C; and 1 g/L fatty acid free BSA). For respiration, cleaned thoracic aorta (2.3–3.0 mg) was initially placed in respiration chambers in respiration media (MiR05) for assessment of baseline respiration. Oxygen flux was measured by addition of glutamate (10 mM) and malate (2 mM) to the chambers in the absence of adenosine diphosphate (ADP) for assessment of state 2 respiration. Once respiration had stabilized, serial additions of ADP were given (7.5 mM total) to stimulate and quantify oxidative phosphorylation (OXPHOS) with electron flux through complex I (state 3, complex I). Complex I and II respiration were assessed by the addition of 10 mM of succinate to the chamber (state 3, complex I + II). Maximal uncoupled respiration was achieved by serial additions of 0.25 µM FCCP (carbonyl cyanide 4-(trifluoromethoxy) phenylhydrazone) (uncoupled). Increases in maximal uncoupled respiration > 20% following the addition of reduced cytochrome c (5 µM) were used for mitochondrial preparation quality control. Rotenone (1 uM) and antimycin A (2.5 uM) were added to assess contributions (inhibitors) of complex I and complex II, respectively.

### Biochemical parameters

Fasted plasma samples from the human cohorts were collected during experimental visits, immediately frozen, and sent to the University of Minnesota Advanced Research and Diagnostic Laboratory for analysis of glucose and lipid profile. For the rodent experiments, plasma samples were collected at sacrifice, immediately frozen, and sent to the Mouse Metabolic Phenotyping Center at University of California (Davis, CA) for glucose, insulin, uric acid, and sodium analyses. Whole blood samples were sent to the Diabetes Diagnostic Laboratory, School of Medicine, University of Missouri (Columbia, MO), for Hemoglobin A1c (HbA1c) analysis. Hematocrit was determined by packed cell volume through centrifugation technique as previously described [37].

### Statistical analysis

Data are presented as mean ± standard error of the mean (SE), as appropriate. Shapiro–Wilk test was performed for assessment of data distribution. Differences in outcomes were determined using unpaired student *t* test, one-way repeated measures ANOVA, or two-way (within-between subjects) repeated measures ANOVA, as appropriate. Holm-Sidak post hoc was performed when significant interactions were found. The Pearson product-moment correlation coefficient was used to examine potential correlations between variables of interest. The results were considered significant when *p* ≤ 0.05. Statistical analyses were performed using GraphPad, Prism (version 9.0). Statistically significant outliers were detected using the ROUT method (*Q* = 5%).

## Results

### Aging is associated with endothelial dysfunction and arterial stiffening

**Fig. 1:**
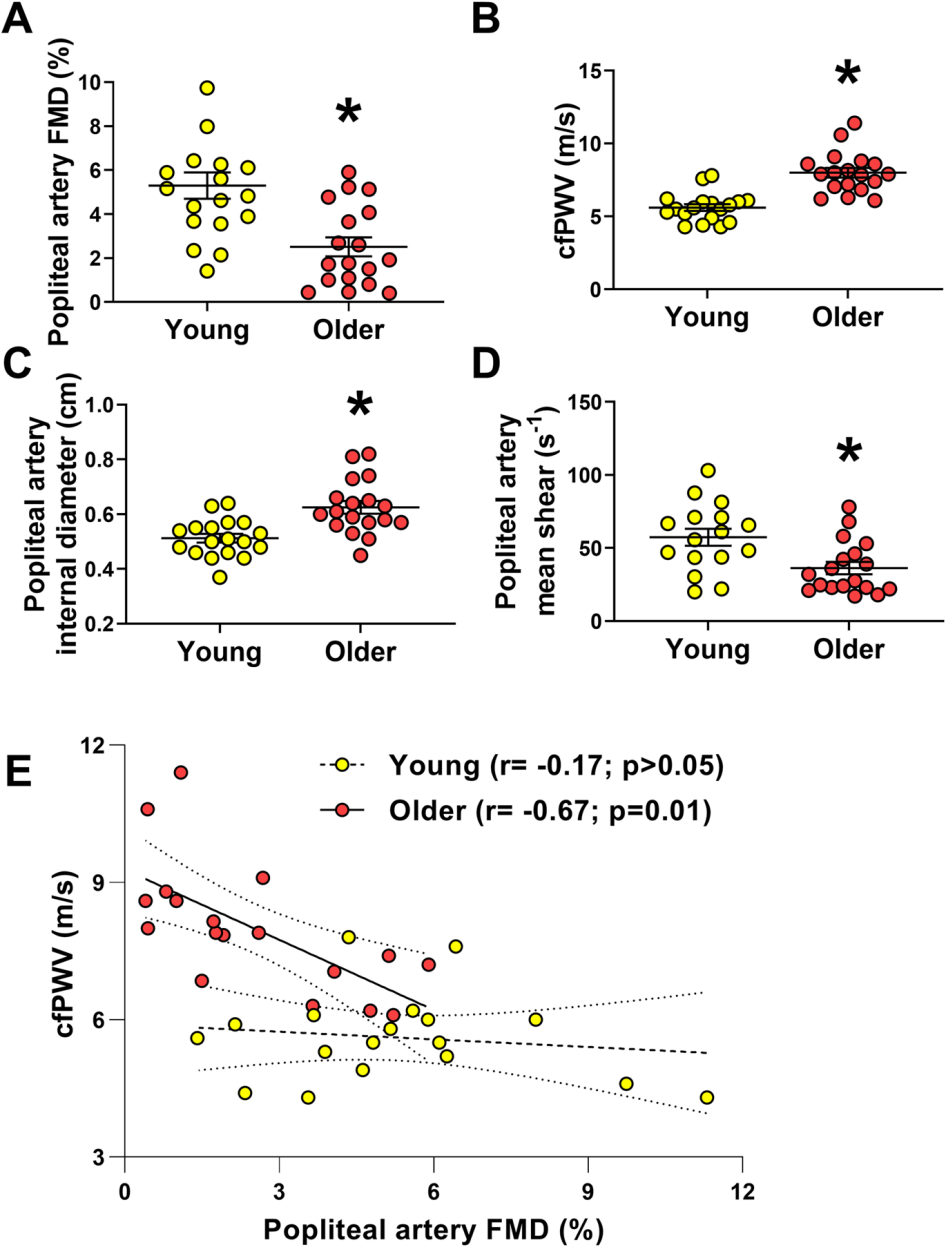
Aging is associated with endothelial dysfunction, increased arterial stiffness, and arterial remodeling in humans. Older humans (*n* = 18) exhibit reduced popliteal artery flow-mediated dilation (**A**), increased vascular stiffness (**B**), increased vascular internal diameter (**C**), and lower vascular shear rate (**D**) compared to younger individuals (*n* = 18). There was a significant negative correlation between PWV and FMD in older individuals, while no significant correlation was found between these variables in their young counterparts (**E**). ROUT identified 2 outliers data points for mean shear data in young group. cfPWV, carotid-femoral pulse wave velocity; FMD, flow-mediated dilation. *p ≤ 0.05 vs. young. Dashed line (–-), linear regression of young group data. Solid line (^**___**^), linear regression of older group data. Unpaired *t* test was performed for all variables comparison

Anthropometric and metabolic characteristics of the younger and older cohort of subjects are presented in Table 1. Older subjects exhibited significantly greater body weight, blood pressure, total and LDL cholesterol, triglycerides, and glucose plasma levels (Table 1). There was no significant difference for plasma HDL between groups (Table 1). Our results, in agreement with previous studies [38, 39], demonstrate that older individuals exhibit reduced endothelial function (diminished popliteal artery FMD) and increased aortic stiffness (faster cfPWV) (Fig. 1A, B), when compared to a cohort of younger adults. Older subjects also displayed greater popliteal artery internal diameter and reduced shear rate (Fig. 1C, D). Males exhibited greater popliteal artery diameter when compared to females; however, no sex-related differences in popliteal FMD, PWV, or shear rate were observed (*supplementary material*). A strong link between endothelial dysfunction and arterial stiffening has been previously reported [40–43]. Endothelial function (popliteal artery FMD) and aortic stiffness (cfPWV) were negatively correlated in older but not in younger individuals (Fig. 1E).

**Table 1:** Characteristics of younger and older subjects

	Young Mean ± SE (*n*)	Older Mean ± SE (*n*)
**Age (years)**	25 ± 1 (18)	61 ± 1 (18)*
**Sex (Female/male)**	9/9	9/9
Race (*n*, %)		
Asian	(1, 5%)	(0, 0%)
Black	(2, 11%)	(0, 0%)
Caucasian/Hispanic	(1, 5%)	(0, 0%)
Caucasian/Non-Hispanic	(14, 77%)	(18, 100%)
Others	(0, 0%)	(0, 0%)
**Height (cm)**	170.06 ± 1.63 (18)	172.64 ± 2.11 (18)
**Body weight (g)**	68.77 ± 2.1 (18)	99.44 ± 4.73 (18)*
**Body mass index (**kg/m^2^**)**	24 ± 1 (18)	33 ± 1 (18)*
**Systolic BP (mmHg)**	119 ± 1 (18)	131 ± 3 (18)*
**Diastolic BP (mmHg)**	74 ± 2 (18)	79 ± 1 (18)
**Fasted blood glucose (mg/dL)**	88.75 ± 1.94 (16)	101.94 ± 2.78 (18) *
**Total cholesterol (mg/dL)**	169.5 ± 8.34 (16)	209.67 ± 10.13 (18) *
**LDL cholesterol (mg/dL)**	100.31 ± 7.89 (16)	133.67 ± 7.71 (18) *
**HDL cholesterol (mg/dL)**	54.43 ± 3.77 (16)	53.67 ± 3.05 (18)
**Triglycerides (mg/dL)**	73.44 ± 7.75 (16)	112.0 ± 10.56 (18) *

*BP* blood pressure. *LDL* low-density lipoprotein. *HDL* high-density lipoprotein.*p < 0.05 vs. control, unpaired *t* test

**Fig. 2:**
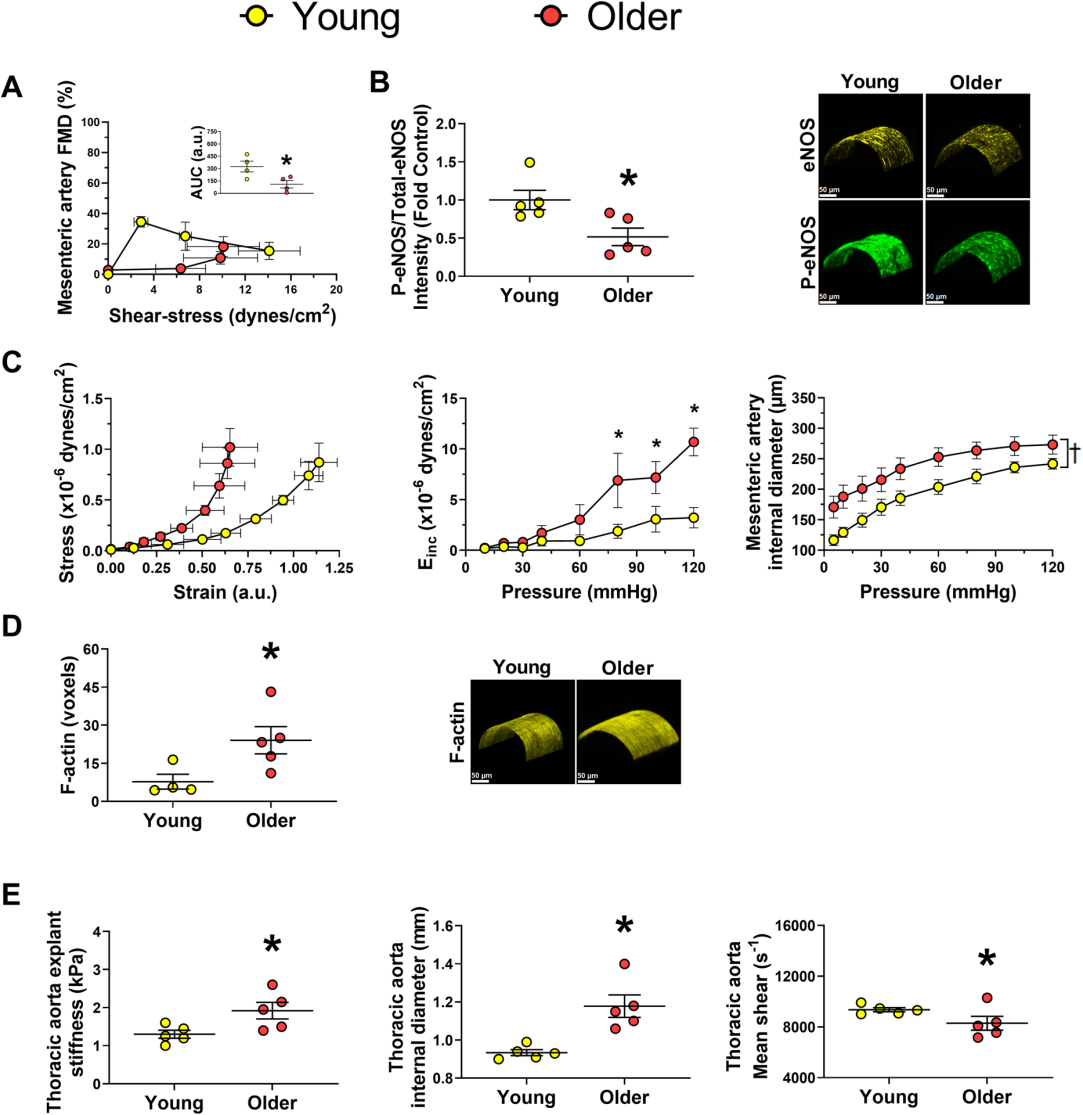
Aged mice exhibit endothelial dysfunction, increased arterial stiffness, and arterial remodeling. Mesenteric arteries of older mice demonstrated reduced endothelium-dependent dilation and eNOS activation (**A, B**). Mesenteric arteries of older mice were also characterized by heightened stiffness, greater internal diameter (**C**), and increased F-actin content (**D**). Thoracic aorta of old C57Bl6/J mice (*n* = 5) demonstrated augmented stiffness, increased vascular internal diameter, and reduced vascular shear rate (**E**) compared to young mice (*n* = 5). ROUT identified 1 outlier data point for F-actin data in young group. AUC, area under the curve; E_inc_, incremental modulus of elasticity; eNOS, endothelial nitric oxide synthase; F-actin, filamentous actin; FMD, flow-mediated dilation; P-eNOS, phosphorylated eNOS *p ≤ 0.05 vs. young; ^†^*p* ≤ 0.05 main effect for group. Unpaired *t* test or two-way ANOVA (group vs condition) following Holm-Sidak for multiple comparisons were performed when appropriate

We then evaluated corresponding measurements of endothelial function and arterial stiffness in a rodent model of aging. Eighty-six-week-old C57Bl6/J male mice had impaired endothelial-dependent vasodilatory function as evidenced by diminished FMD when expressed as percent and absolute change in diameter (Fig. 2A and *supplementary material*, respectively) and eNOS activation (Fig. 2B) in mesenteric arteries. Mesenteric arteries from older mice also had blunted relaxation responses to SNP (*supplementary material*). Additionally, the mesenteric arteries of older mice exhibited heightened stiffness, as shown by the strain–stress curves and the incremental modulus of elasticity and increased internal passive diameter (Fig. 2C). No significant differences in mesenteric artery constrictor responses to KCl or Phe, external diameter, or cross-sectional area were evidenced between groups (*supplementary material*). As arterial stiffening is associated with increased polymerization of actin [44], we assessed F-actin content in mesenteric arteries. Aging was associated with increased F-actin content (Fig. 2D). Similarly, older C57Bl6/J male mice also demonstrated augmented ex vivo aortic stiffness, increased thoracic aorta diameter, and reduced shear rate (Fig. 2E), reinforcing the appropriateness of this mouse model as a preclinical model of human vascular aging.

### Six weeks of SGLT2 inhibition did not affect body weight, blood pressure or glucose metabolism

As shown in Table 2, neither blood pressure nor body weight were significantly affected by SGLT2 inhibition. Additionally, SGLT2 inhibition did not significantly impact glucose tolerance, as represented by the AUC of plasma glucose concentration during the 120-min glucose tolerance test (Table 2 and *supplementary material*). At sacrifice, samples for plasma glucose, insulin, HbA1c, sodium, uric acid, and hematocrit were obtained. No significant differences were seen between cohorts (Table 2).

**Table 2:** Body weight at sacrifice, blood pressure, and other biochemical parameters

	Control Mean ± SE (*n*)	Empa Mean ± SE (*n*)
Body weight (g)	38 ± 0.8 (19)	38 ± 1.0 (19)
Systolic blood pressure (mmHg)	106 ± 1 (10)	107 ± 2 (10)
Fed glucose (mg/dL)	205 ± 5 (9)	204 ± 5 (9)
GTT, glucose-AUC_0-120 min_ (a.u)	36,441 ± 936 (9)	35,070 ± 1355 (10)
Fed insulin (pg/mL)	1642 ± 199 (9)	1487 ± 304 (9)
Hemoglobin A1c (%)	4.18 ± 0.1 (10)	4.13 ± 0.1 (8)
Uric acid (mg/dL)	1.6 ± 0.1 (9)	1.5 ± 0.1 (9)
Sodium (mEq/L)	150 ± 0.4 (9)	150.9 ± 0.7 (9)
Hematocrit (%)	43 ± 0.7 (10)	42 ± 0.9 (10)
Malondialdehyde (μmol/L)	4.7 ± 0.2 (9)	3.5 ± 0.1 (9)*

*GTT* glucose tolerance test. *AUC* area under the curve. *p < 0.05 vs. control, unpaired *t* test

### SGLT2 inhibition improves endothelial function, attenuates arterial stiffness, and reduces actin stress fibers in mesenteric arteries

**Fig. 3:**
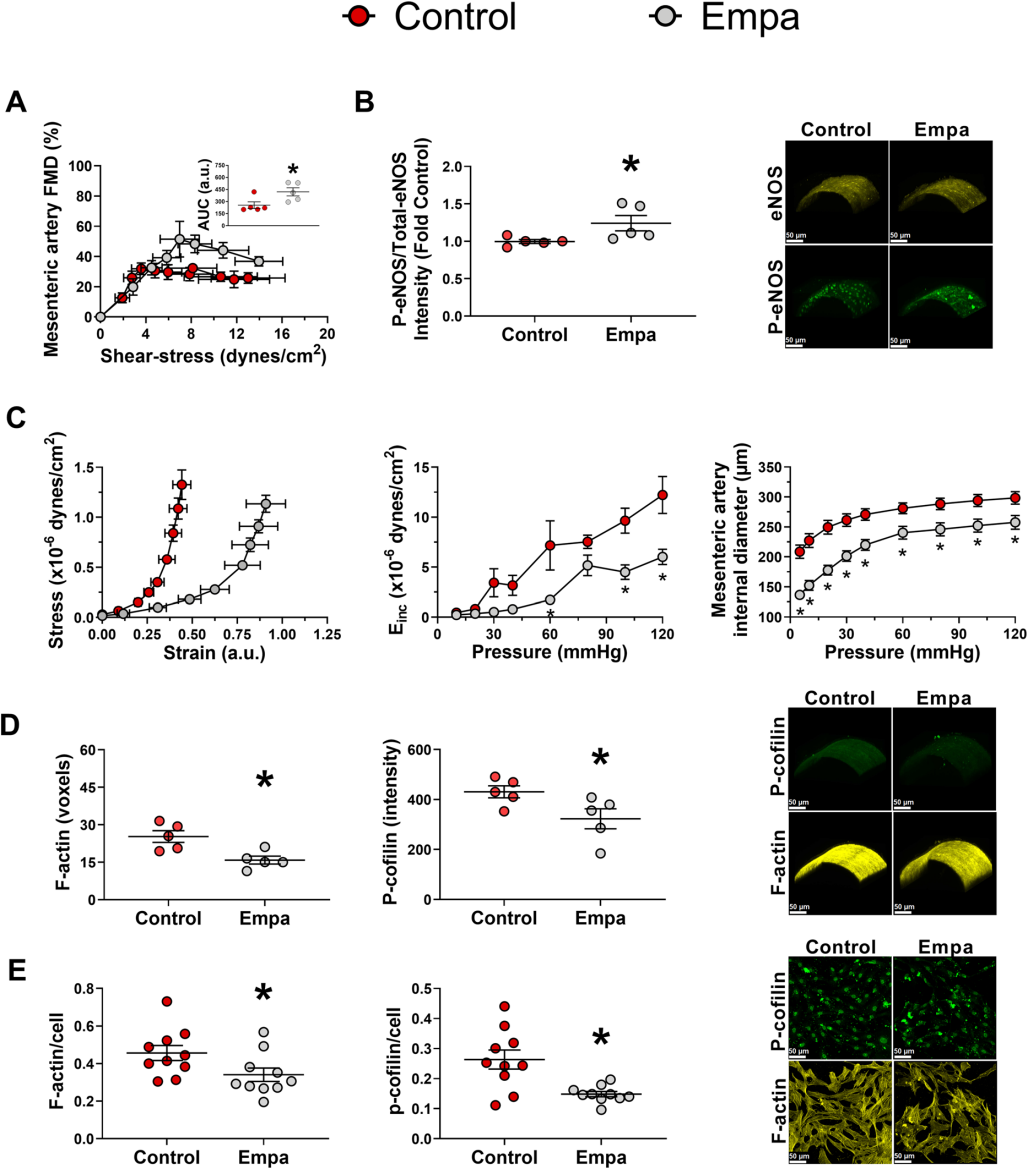
SGLT2 inhibition improves endothelial function and diminishes mesenteric artery stiffness and internal diameter in aged mice. Empa also decreases F-actin content and cofilin phosphorylation in mesenteric artery of aged mice and in human coronary VSMCs. Empa-treated mice (*n* = 5) exhibited greater mesenteric artery FMD (**A**) and eNOS phosphorylation (**B**) than controls (*n* = 5). Mice treated with Empa had lesser arterial stiffness (Empa, *n* = 9; control, *n* = 9), smaller mesenteric arteries internal diameters (Empa, *n* = 9; control, *n* = 9) (**C**), and reduced F-actin (Empa, *n* = 5; control, *n* = 5) and P-cofilin content (**D**) compared to control (Empa, *n* = 5; control, *n* = 5). VSMCs were treated for 24 h with Empa (*n* = 10) or vehicle (control, *n* = 10) (**E**). Empa-treated VSMCs showed reduced F-actin content compared to control (**E**). Treatment with Empa was associated with decreased P-cofilin compared to control (**E**). AUC, area under the curve; eNOS, endothelial nitric oxide synthase; Empa, empagliflozin; F-actin, filamentous actin; FMD, flow-mediated dilation; P-cofilin, phosphorylated cofilin; P-eNOS, phosphorylated eNOS. *p ≤ 0.05 vs control. ^†^*p* ≤ 0.05 main effect for group. Unpaired *t* test or two-way ANOVA (group vs condition) following Holm-Sidak for multiple comparisons were performed when appropriate

Aging is characterized by impairments in vascular function. As depicted in **Fig. ****3**, Empa treatment improved endothelium-dependent vasodilation as reflected by an increase in mesentery artery FMD when expressed as percent and absolute change in diameter (Fig. 3A and supplementary material, respectively). This improvement was accompanied by augmented eNOS activation (Fig. 3B). No between-groups differences were found for constrictor responses to KCl and Phe or the vasodilatory response to the endothelium-independent dilator SNP (*p* > 0.05) (supplementary material).

We and others have shown that endothelial dysfunction is associated with arterial stiffening (Fig. 1) [40, 45, 46]. Empa treatment resulted in attenuation in arterial stiffness (Fig. 3C). These findings paralleled with reductions in mesentery artery internal (Fig. 3C) and external diameters (supplementary material) without changes in elastin/collagen content (supplementary material). As older mice exhibit increased content of F-actin in mesenteric arteries (Fig. 1), we examined the impact of SGLT2 inhibition on F-actin content. Empa decreased F-actin content in the mesenteric arteries of 80-week-old C57BL6/J mice (Fig. 3D). Accordingly, this was accompanied by lessened P-cofilin content (Fig. 3D). Cofilin is an actin modulatory enzyme that severs actin filaments, and its phosphorylation results in its inactivation [47]. To determine if the previous results were related to a direct effect of the SGLT2 inhibition on the vascular wall, we incubated VSMC with Empa 500 nM for 24 h. We report that Empa incubation also lowered F-actin and P-cofilin content in cultured VSMC (Fig. 3E).

### SGLT2 inhibition alleviates thoracic aorta stiffness, decreases thoracic aorta diameter, and increases vascular shear rate

**Fig. 4:**
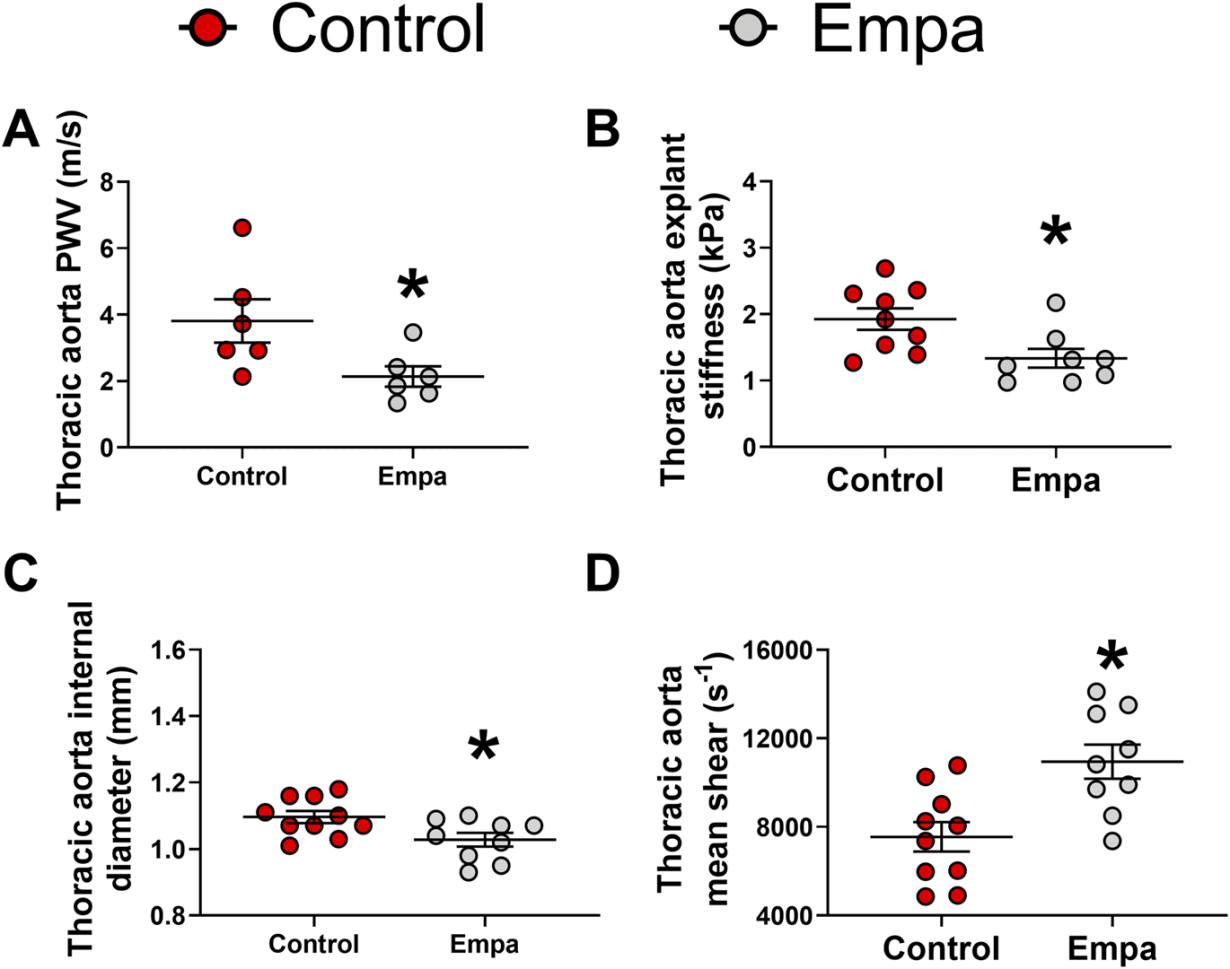
Six weeks of SGLT2 inhibition decreases aortic stiffening and remodeling in aged mice. Empa-treated mice had lower aortic PWV (Empa, *n* = 6; control, *n* = 6) (**A**) and aortic explant stiffness (Empa, *n* = 8; control, *n* = 9) (**B**) when compared to controls. Empa treatment was also associated with reduced thoracic aorta internal diameter (Empa, *n* = 9; control, *n* = 10) (**C**) and greater mean shear (Empa, *n* = 9; control, *n* = 10) compared to controls (**D**). ROUT identified 3 outliers data points in total (1 aortic explant stiffness, Empa; 1 thoracic aorta internal diameter, Empa; 1 mean shear, Empa). Empa, empagliflozin. *p ≤ 0.05 vs control. Unpaired *t* test was performed for all variables comparison

The aging-related increase in aortic diameter and reduction in shear stress occurs in parallel with endothelial dysfunction and changes in arterial wall composition, two major contributors to aortic stiffening [39, 48–50]. SGLT2 inhibition for 6 weeks reduced thoracic aorta stiffness when compared with controls (Fig. 4A, B). Additionally, SGLT2 inhibition decreased internal aortic diameter and significantly increased mean shear rate compared with the control group (Fig. 4C, D). However, immunohistochemical assessment of aortic collagen and elastin content did not reveal any differences between the cohorts (supplementary material).

### SGLT2 inhibition reduces circulating MDA and downregulates pathways involved in vascular reactive oxygen biosynthesis

**Fig. 5:**
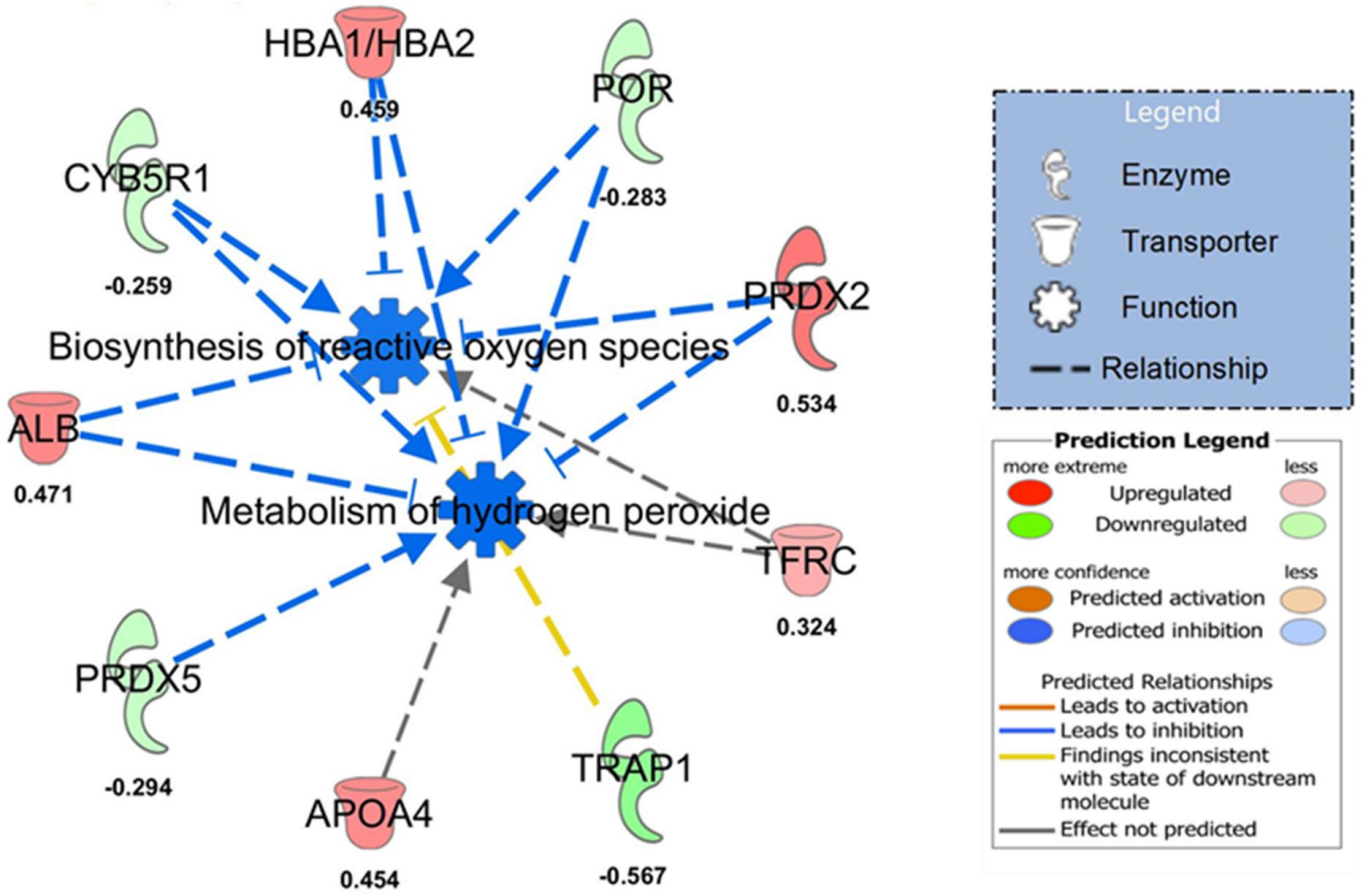
Proteomic analysis of thoracic aorta from control and Empa-treated old mice. Empa treatment is associated with downregulation of pathways linked to biogenesis of reactive oxygen species and metabolism of hydrogen peroxide. ALB, albumin; APOA4, Apolipoprotein A-IV; CYB5R1, NADH-cytochrome b5 reductase 1; HBA1/HBA2, hemoglobin subunit alpha 1/2; POR, NADPH-cytochrome P450 reductase; PRDX2, peroxiredoxin-2; PRDX5, peroxiredoxin-5; TFRC, transferrin receptor protein 1; TRAP1, Heat shock protein 75 kDa mitochondrial. Data expressed as fold change relative to control. Empa, *n* = 10; and control, *n* = 10. Unpaired *t* test was performed for all variables comparison (please see supplementary material for further description of the statistical analysis)

Oxidative stress has been associated with the genesis of aging-related endothelial dysfunction and increased arterial stiffness [51–53]. Herein, we show that SGLT2 inhibition results in lower plasma MDA levels compared to controls (Table 1). We then examined known sources of reactive oxygen species. No difference in aorta mitochondrial function was found between cohorts (supplementary material). However, the proteomic analysis identified 192 proteins that were differentially expressed in the aortas of Empa-treated mice. Among them, unbiased ingenuity pathway analysis detected top expression changes in signaling pathways involved in lipid and carbohydrate metabolism and connective tissue disorders (Fig. 5). Of particular interest, we found that SGLT2 inhibition resulted in downregulation of key proteins (0.285-fold change in xanthine dehydrogenase/oxidase expression) and pathways involved in reactive oxygen species biosynthesis (Fig. 5).

## Discussion

To our knowledge, this is the first study to examine the potential role of SGLT2 inhibition in reversing vascular aging. Endothelial dysfunction and arterial stiffening are key events in the pathogenesis of CVD in older adults, and here, we report that these two variables are negatively correlated. We then provide evidence that 6 weeks of SGLT2 inhibition with Empa decrease endothelial dysfunction and attenuate arterial stiffness in aged mice with established vascular dysfunction. These changes were paralleled with decreased passive arterial diameters and VSMC content of F-actin and P-cofilin. In addition, we demonstrated decreased circulating markers of oxidative stress and lowered vascular expression of xanthine oxidase with Empa.

Endothelial dysfunction and arterial stiffening are progressive and interconnected occurrences characteristic of vascular aging. Accordingly, our findings in young and older adults confirm previous clinical data demonstrating the impact of aging on endothelial function and arterial stiffness [54]. Importantly, our rodent model of aging recapitulates these human findings by exhibiting endothelial dysfunction and increased arterial stiffness. Our data also demonstrated a significant inverse relationship between FMD and PWV in older, but not young, individuals. These findings support the notion that endothelial dysfunction may be a determinant of aging-related arterial stiffening and reinforce the existence of a crosstalk between these two variables in individuals at high risk for CVD [55, 56]. In this context, the current investigation reveals that 6 weeks of SGLT2 inhibition improved endothelial function and decreased arterial stiffness in aged mice. Additionally, our findings show that these vascular effects were independent of changes in blood pressure, body weight, and glucose control. In relation to the later, our results mimic those of large clinical trials in which Empa treatment reduced the risk for CVD independently of blood glucose control in heart failure patients with and without type 2 diabetes [16, 57–59].

Systemic oxidative stress is characteristic of aging [60, 61]. In the vasculature, aging-associated oxidative stress is a major cause of decreased nitric oxide bioavailability, endothelial dysfunction, and arterial stiffening [39, 62–64]. Empa treatment decreased circulating levels of malondialdehyde, a marker of lipid peroxidation, in old C57Bl6/J male mice. In addition, our proteomic analysis revealed downregulation of pathways linked to biosynthesis of reactive oxygen species and metabolism of hydrogen peroxide in the aortas of Empa-treated mice. Previous preclinical studies in a diabetes model have shown that SGLT2 inhibition ameliorates oxidative stress in renal, cardiac, and vascular tissues [65–67]. Similarly, Solini et al. demonstrated that a 2-day intervention with the SGLT2 inhibitor dapagliflozin in subjects with type 2 diabetes improved brachial artery FMD and decreased PWV. These changes were independent of blood pressure and glycemic changes and were seen in parallel with a decrease in circulating markers of oxidative stress [68].

Our proteomic analysis also revealed decreased expression of xanthine oxidase in the aortas of mice treated with Empa when compared to controls. Xanthine oxidase is a source of superoxide and hydrogen peroxide in aging-associated oxidative stress [52, 69]. Mitochondria is a major target of oxidative damage and also a primary source of reactive oxygen species in the aged organism [70, 71]. Given that, we also examined the role of mitochondrial dysfunction as a source of increased reactive oxygen species. However, in our model, SGLT2 inhibition did not have an impact on vascular mitochondrial function. Thus, it is possible that the beneficial vascular effects of SGLT2 inhibition in our model of vascular aging are related to decreased systemic oxidative stress combined with reduced vascular reactive oxygen species production. In this regard, reduced vascular reactive oxygen species production would favor eNOS coupling, thereby improving endothelial function, increasing nitric oxide bioavailability, and consequently attenuating arterial stiffness.

Changes in blood viscosity and blood pressure have also been described as potential mechanisms behind the positive effects of SGLT2 inhibitors on the vasculature [72, 73]. However, in the present work, SGLT2 inhibition was not associated with significant changes in blood pressure or hematocrit, a main determinant of blood viscosity [74]. Instead, we found that mice treated with Empa had reduced aortic and mesenteric arterial diameter and consequently increased vascular wall shear stress and eNOS phosphorylation. Considering this scenario, we posit that the de-stiffening effect of Empa may also be linked to improvements in vessel wall shear stress. Vessel wall shear stress is an important hemodynamic phenomenon associated with nitric oxide endothelium-dependent function and stiffness that is weakened with aging due to excessive enlargement of the vasculature [48, 75]. Thus, it is possible that wall shear stress-driven improvement in endothelial function may partially explain the Empa-induced de-stiffening effect.

It is well known that arterial stiffening is a strong predictor of cardiovascular morbidity and mortality independent of traditional cardiovascular risk factors [76–78]. Indeed, aging is accompanied with changes in vascular structures associated with increased stiffness. Specifically, the paradigm of aging-related progression of arterial stiffness has recently shifted beyond that of elastin/collagen content and has also included VSMC stiffness as a determinant factor of arterial stiffening in older individuals [79, 80]. Here, we report that older mice treated with SGLT2 inhibition exhibited reduced arterial stiffness without changes in elastin/collagen vessel wall content. Nevertheless, the reduced stiffness in mesenteric arteries was associated with decreased P-cofilin and F-actin content. Likewise, VSMCs treated with Empa showed reduced P-cofilin and F-actin content when compared to control. Increased actin polymerization has been previously described in a rodent model of aging and cellular stiffness [81]. Moreover, our group has recently demonstrated that reduction of P-cofilin and F-actin results in decreased arterial stiffness [82]. Consequently, our results support a direct effect of SGLT2 on VSMC as an additional de-stiffening mechanism in this model of aging. However, it has been shown that an oxidative stress milieu decreases the ability of cofilin to sever actin filaments resulting in cytoskeleton stiffening [83, 84]. Thus, it is possible that the reduction in cofilin phosphorylation and F-actin content found in the mesenteric arteries of older mice treated with Empa was also driven by decreases in reactive oxygen species.

Several aspects of the present investigation warrant further consideration. In line with current population trends [85, 86], our older human cohort displayed a clustering of cardiovascular disease risk factors which likely contributed to the heightened endothelial dysfunction and arterial stiffening associated with aging. Further, we speculate that the existence of a negative correlation between FMD and PWV in the older cohort underscores the central role of nitric oxide bioavailability in modulating arterial mechanics in the setting of aging-related cardiovascular and metabolic abnormalities. In other words, variations in endothelial function in such a lower range as observed in the older cohort may be a critical determining factor of arterial stiffening in this population. Similar to our findings, a previous investigation reported that endothelial dysfunction is a determinant of aortic stiffness only in subjects with hypertension and type 2 diabetes but not in hypertensive patients without diabetes [56]. Along these lines, it is conceivable that young individuals with lower endothelial function may be predisposed to greater arterial stiffening with aging compared to young subjects with high endothelial function. However, this hypothesis remains to be tested.

Another important consideration is that, in humans, FMD was assessed in conduit arteries, whereas in mice, it was assessed in resistance arteries. This should be acknowledged because the role of shear stress as a key regulator of vascular tone varies across vascular beds [87]. Similarly, we only analyzed the impact of SGLT2 inhibition in cultured human coronary VSMCs is conceivable that cultured VSMCs from mouse mesenteric arteries may exhibit a different response.

In the present investigation, we did not assess blood pressure in the young cohort of mice. Previous work has shown modest elevations in blood pressure throughout the lifetime of the C57Bl6 mice [88]. Notably, additional interventions are commonly needed to elicit a frank hypertensive phenotype in C57Bl6 [89]. In fact, it is intriguing that such outward remodeling as seen in the current older mice cohort did not cause a reduction in blood pressure. Perhaps, the lack of outward remodeling-induced reduction in blood pressure reflects activation of renal counterregulatory mechanisms [90] or is offset by the reduced endothelial function and augmented arterial stiffness shown in the older cohort. In connection to this, in our current work, 6 weeks of SGLT2 inhibition did not impact blood pressure in older mice. Equally, a previous study showed that SGLT2 inhibition had no blood pressure lowering effects in normotensive diabetic patients [91]. Given the above, it can be postulated that the lack of changes in blood pressure in response to SGLT2 inhibition is related to the absence of a hypertensive phenotype in our mouse model of aging. Lastly, it is important to acknowledge that we only examined the impact of SGLT2 inhibition in a male mouse model of aging. Even though this rodent model mimics the vascular findings documented in our cohort of women and men, future experiments should be conducted in aged female mice.

The findings of the present investigation provide evidence that aging-related endothelial dysfunction and arterial stiffening are significantly ameliorated in a mouse model of aging upon treatment with SGLT2 inhibition. Specifically, a 6-week treatment with Empa resulted in greater eNOS phosphorylation and downregulation of vascular P-cofilin, F-actin, and expression of proteins associated with production of reactive oxygen species. Even though the impact of aging on vascular expression and function of SGLT2 are still unclear, the current findings stimulate the need for further clinical investigations to determine the potential role of SGLT2 inhibition as a therapeutic tool to delay or reverse vascular aging. Finding alternative approaches to treat and prevent CVD-related death in older individuals will be crucial as the population of adults aged 65 years and older is expected to grow an additional 44% by 2030 [1]. Given that, a significant increase in the number of deaths associated with CVD can be projected [1, 2].

## Supplementary Information

Below is the link to the electronic supplementary material.

Supplementary file1 (DOCX 927 KB)

## Data Availability

The data underlying this article will be shared on reasonable request to the corresponding author.
