# Supplementary material for: SGLT2 inhibition attenuates arterial dysfunction and decreases vascular F-actin content and expression of proteins associated with oxidative stress in aged mice
Source: GeroScience. 2022 Apr 15;44(3):1657–75. doi: 10.1007/s11357-022-00563-x (PMC9213629; doi:10.1007/s11357-022-00563-x)
Supplement: Supplementary file 1 — Supplementary file1 (DOCX 927 KB) [file 11357_2022_563_MOESM1_ESM.docx]

**SUPPLEMENTARY MATERIAL**

**
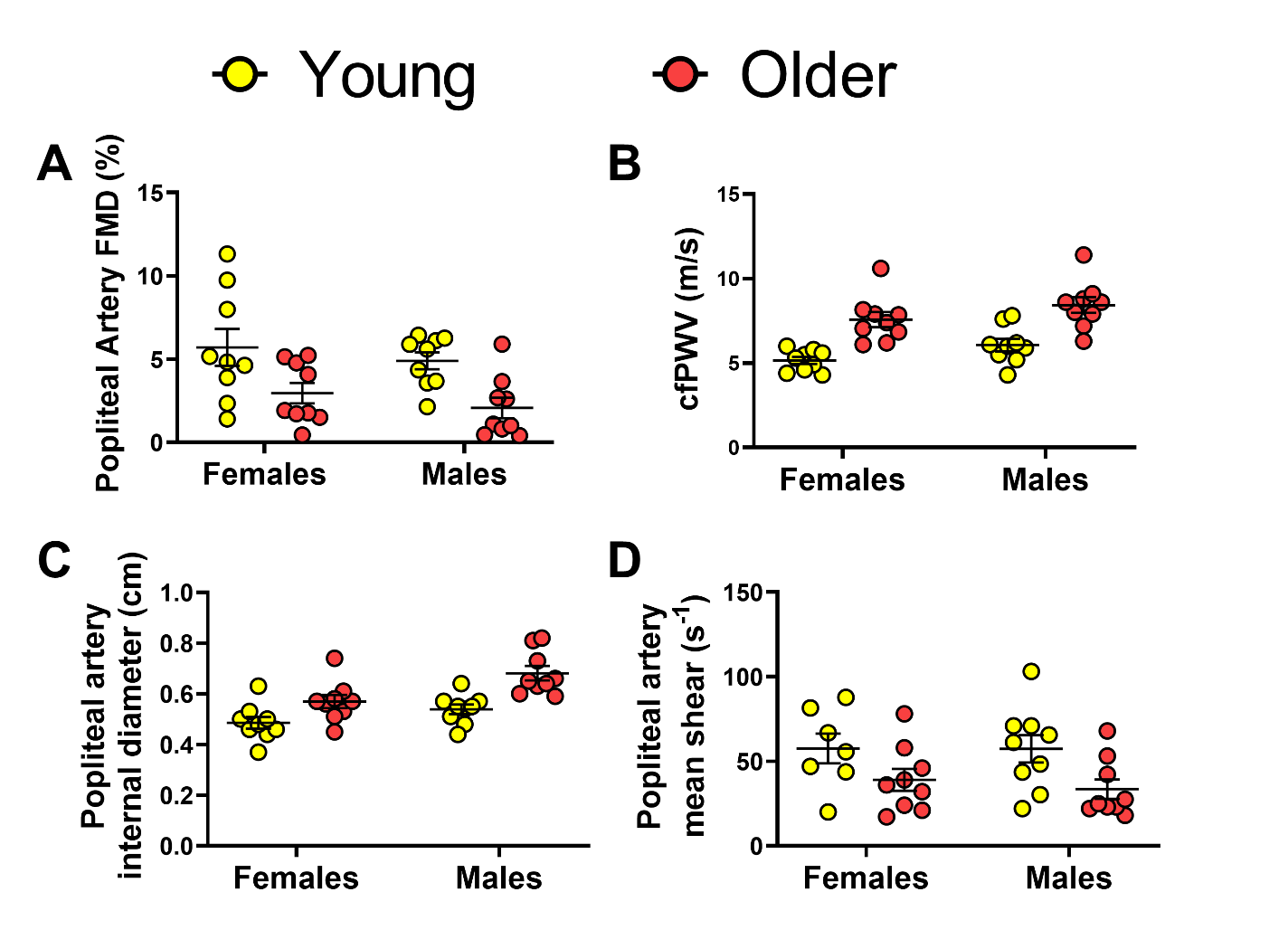
**

**Fig. S1. Aging is associated with popliteal artery endothelial dysfunction, arterial stiffening, increased internal diameter, and reduced shear rate in females and males.** Older (females, n=9; males. N=9) individuals exhibited reduced popliteal artery flow-mediated dilation (FMD) as well as augmented arterial stiffness when compared to young (females, n=9; males, n=9) individuals (**A).** (**B**). Popliteal artery of older (females, n=9; males, n=9) individuals showed greater internal diameter when compared to young ones (females, n=9; males. N=9) (**C**). Additionally, older (females, n=9; males. N=9) individuals exhibited reduced popliteal artery mean shear compared to young (females, n=7; males, n=9) (**D**). ROUT identified 2 outliers data points for popliteal artery mean shear (2 young females). Two-way ANOVA (group vs condition) was performed. There was a significant age effect (p<0.05) for all variables. No significant interactions were found.

**
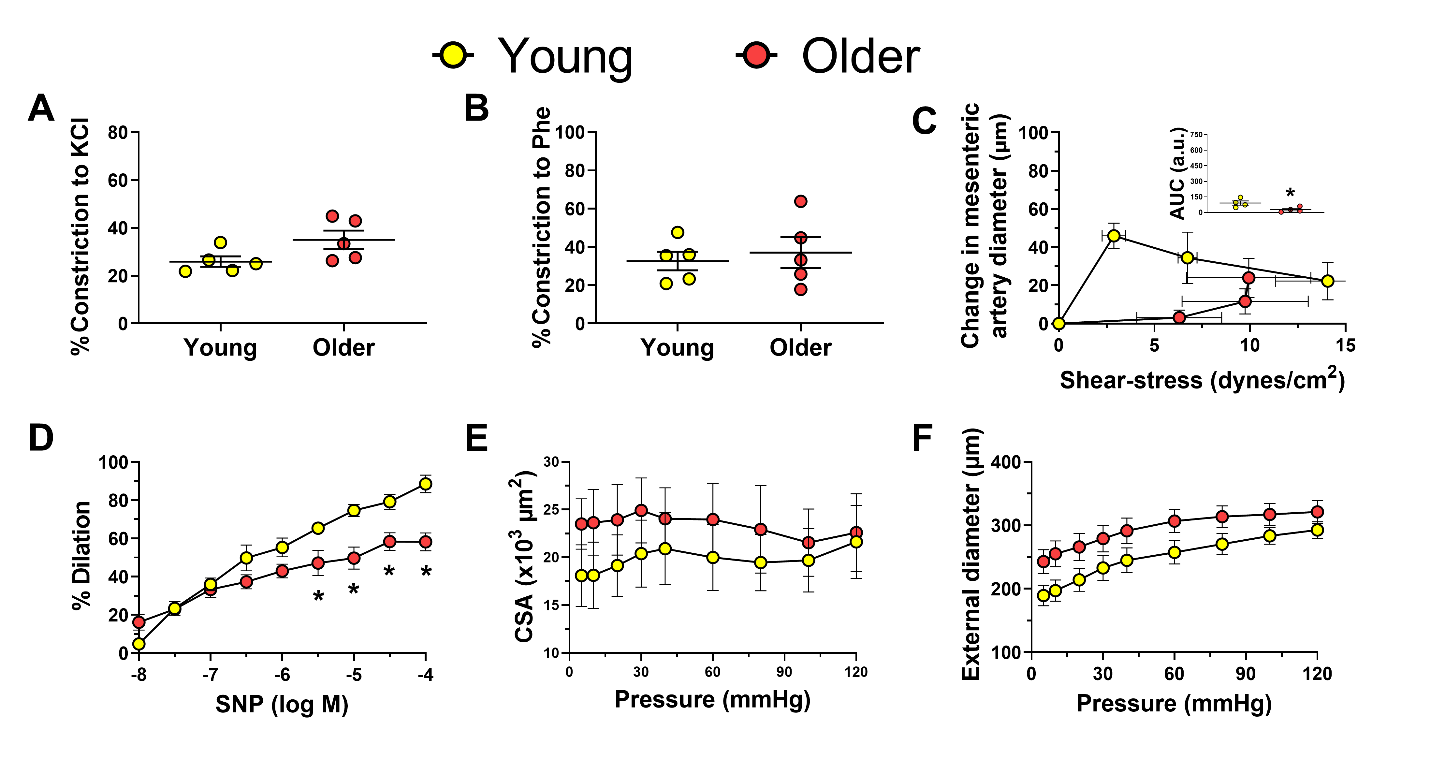
**

**Fig. S2. Aging is associated with mesenteric artery endothelium-dependent and -independent vasomotor dysfunction without differences in external artery diameter or cross-sectional area.** No significant differences between young (n= 5) and older (n=5) mice in mesenteric artery constrictor responses to potassium chloride (KCl) or phenylephrine (Phe) were seen (**A,B**). Mesenteric arteries of older mice exhibited reduced flow-mediated dilation (FMD) normalized to shear-stress (young, n=4; older, n=4) as well as impaired vasodilation in response to sodium nitroprusside (SNP) (young, n=5; older, n=5) compared to young mice (**C, D**). No significant differences in cross-sectional area (CSA) (**E**) or external diameter (**F**) were found between young (n=5) and older (n=5) mice. ROUT identified 2 outliers data points for mesenteric artery FMD data (1 young; 1 older). *, p≤0.05 vs young. Unpaired T-test or Two-way ANOVA (group vs condition) following Holm-Sidak for multiple comparisons were performed when appropriate.


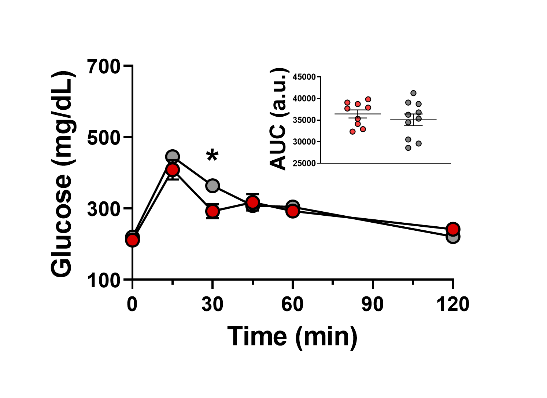


**Fig. S3. Glucose excursions in response to an intraperitoneal dextrose injection (1 g/Kg BW) in control and Empa-treated mice.** There were no significant differences in plasma glucose concentration at 0, 15, 45, and 120 min after the dextrose injection between control (n= 10) and Empa-treated (n=9) mice. Post hoc analysis showed that glucose plasma concentration of the Empa-treated mice at 30 min was significantly lower than control. No significant differences in the area under the curve of the glycemic response were found between groups. Unpaired T-test or Two-way ANOVA (group vs condition) following Holm-Sidak for multiple comparisons were performed when appropriate. ROUT identified 1 outlier (control group).


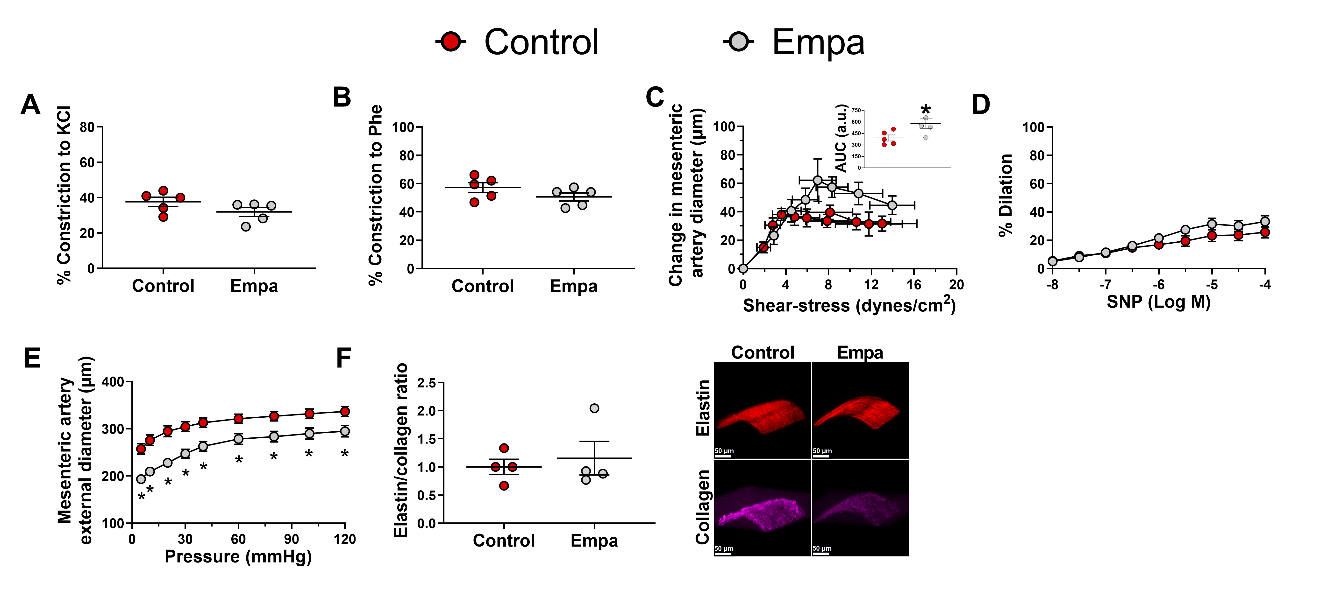


**Fig. S4. Six weeks of SGLT2 inhibition improves mesenteric artery endothelial function without impacting endothelium-independent dilation. Empa also decreases mesenteric artery external diameter but has no effect on elastin/collagen content.** There were no significant differences between control (n= 5) and Empa-treated (n=5) mice in the mesenteric artery constrictor responses to potassium chloride (KCl) and phenylephrine (Phe) (**A,B**). Empa treatment was associated with improved mesenteric artery flow-mediated dilation (FMD) normalized to shear-stress (Empa, n=5; control, n=5) (**C**) while no differences in mesenteric artery responses to the endothelium-independent dilator sodium nitroprusside (SNP) (Empa, n=5; control, n=5) (**D**) were found. Empa reduced mesenteric artery external diameter (Empa, n=5; control, n=5) (**E**), however had no impact on elastin/collagen content (Empa, n=4; control, n=4) (**F**). Empa, empagliflozin. Unpaired T-test or Two-way ANOVA (group vs condition) following Holm-Sidak for multiple comparisons were performed when appropriate.


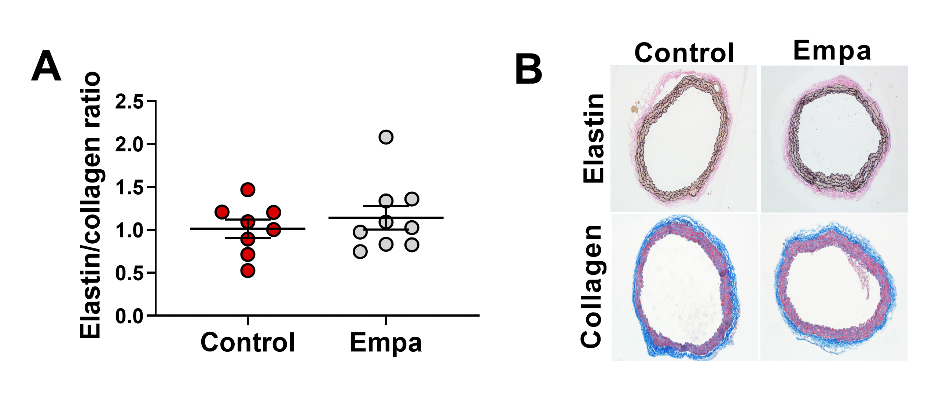


**Fig. S5**. **SGLT2 inhibition for six-weeks does not alter aortic elastin/collagen content.** Empa treatment (n=8) did not change elastin/collagen content in thoracic aorta compared to control (n=9) (**A,B**). Empa, empagliflozin. Unpaired t-test was performed.

**
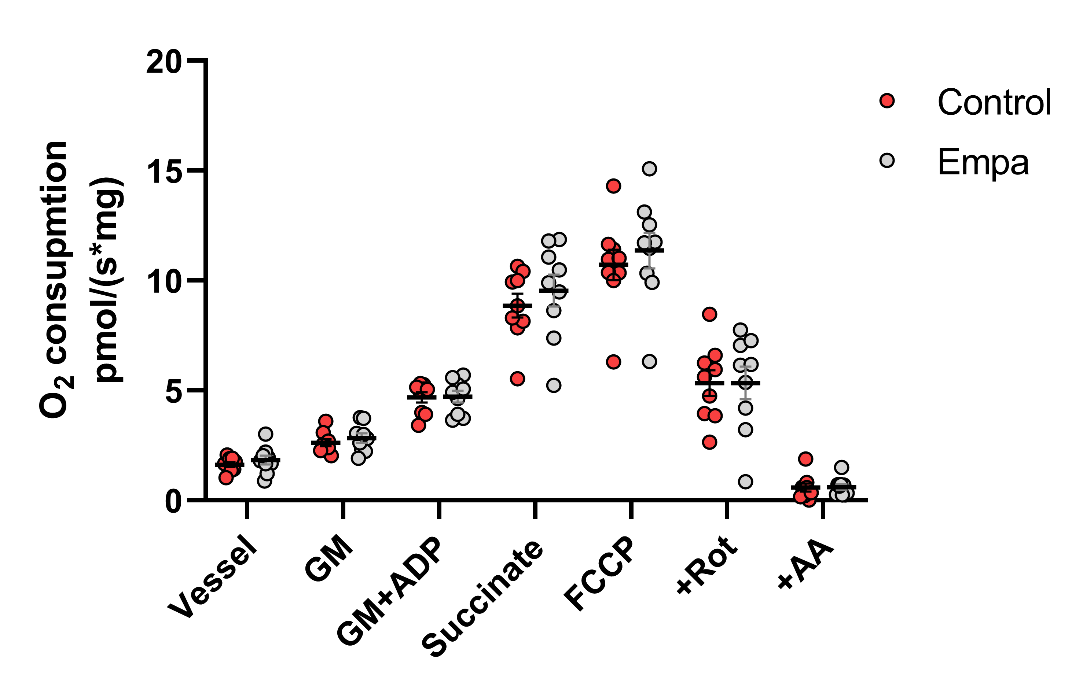
**

**Fig. S6. Six weeks of SGLT2 inhibition treatment does not impact aortic mitochondrial function in aged mice.** +AA, antimycin A; +Rot, rotenone; GM, glutamate + malate; GM+ADP, glutamate + malate + adenosine diphosphate, FCCP, Carbonyl cyanide 4-(trifluoromethoxy) phenylhydrazone. Empa, n=9 and control, n=9. Two-way ANOVA was performed for group vs condition comparisons.

**Proteomics analysis**

*Spectral library built-up*: For spectral library, 10ug digested peptides from each sample were combined and conducted high pH fractionation. Total 8 fractions were collected and used for spectra library set up. The final peptide and protein FDR was filtered to 1%. Total 45671 ions, 34959 peptides and 5096 protein groups were included in the library.

*DIA data*: Total ~4200 proteins were identified. The DIA data were filtered with precursor FDR and protein group FDR to 1%, at least a protein was identified in five samples(reps) per group (total 10 samples(reps)), at least protein intensity >1000 in one sample. On average ~3700 proteins were identified/quantified in each sample.

*Statistical analysis*: t-test was applied for control and Empa group comparisons. After filtering by p value 0.05, fold change 1.2-fold, 192 proteins showed significantly change.

**Table S1. Proteomics C57Bl6/J mice: Empa vs Control**

| **Gene** | **Protein** | **Fold** | **p-value** | |
| --- | --- | --- | --- | --- |
| Hspa12a | Heat shock 70 kDa protein 12A | -0.3 | | <0.01 |
| Txnl4a | Thioredoxin-like protein 4A | -0.4 | | <0.01 |
| Uap1 | UDP-N-acetylhexosamine pyrophosphorylase | -0.3 | | <0.01 |
| Ccar1 | Cell division cycle and apoptosis regulator protein 1 | -0.3 | | <0.01 |
| Hspa12b | Heat shock 70 kDa protein 12B | -0.3 | | <0.01 |
| Epb41l2 | Band 4.1-like protein 2 | -0.3 | | <0.01 |
| Acly | ATP-citrate synthase | -0.6 | | <0.01 |
| Xdh | Xanthine dehydrogenase/oxidase | -0.3 | | <0.01 |
| Syde1 | Rho GTPase-activating protein SYDE1 | -0.3 | | <0.01 |
| Adh1 | Alcohol dehydrogenase 1 | -0.3 | | <0.01 |
| Cpsf4 | Cleavage and polyadenylation specificity factor subunit 4 | -0.4 | | <0.01 |
| Rida | 2-iminobutanoate/2-iminopropanoate deaminase | 0.5 | | <0.01 |
| Ddx46 | Probable ATP-dependent RNA helicase DDX46 | -0.3 | | <0.01 |
| Rps14 | 40S ribosomal protein S14 | -0.3 | | 0.01 |
| Pycr2 | Pyrroline-5-carboxylate reductase 2 | -0.3 | | 0.01 |
| Nt5c3a | Cytosolic 5'-nucleotidase 3A | 0.5 | | 0.01 |
| Arpc1a | Actin-related protein 2/3 complex subunit 1A | 0.3 | | 0.01 |
| Mri1 | Methylthioribose-1-phosphate isomerase | 0.4 | | 0.01 |
| Serinc3 | Serine incorporator 3 | 0.4 | | 0.01 |
| Masp1 | Mannan-binding lectin serine protease 1 | 0.4 | | 0.01 |
| Mrc1 | Macrophage mannose receptor 1 | -0.3 | | 0.01 |
| Clec10a | C-type lectin domain family 10 member A | -0.5 | | 0.01 |
| Ftl1 | Ferritin light chain 1 | -0.3 | | 0.01 |
| Luc7l3 | Luc7-like protein 3 | -0.4 | | 0.01 |
| Ubac1 | Ubiquitin-associated domain-containing protein 1 | 0.5 | | 0.01 |
| Metap1 | Methionine aminopeptidase 1 | -0.3 | | 0.01 |
| Rer1 | Protein RER1 | -0.3 | | 0.01 |
| C2 | Complement C2 | 0.4 | | 0.01 |
| Rps13 | 40S ribosomal protein S13 | -0.5 | | 0.01 |
| Cryab | Alpha-crystallin B chain | -0.3 | | 0.01 |
| Thrsp | Thyroid hormone-inducible hepatic protein | -1.2 | | 0.01 |
| Acaca | Acetyl-CoA carboxylase 1 | -0.8 | | 0.01 |
| Fasn | Fatty acid synthase | -1.0 | | 0.01 |
| Dglucy | D-glutamate cyclase, mitochondrial | -0.3 | | 0.01 |
| Taok3 | Serine/threonine-protein kinase TAO3 | 0.5 | | 0.01 |
| Gypc | Glycophorin-C | 0.8 | | 0.01 |
| Slc25a1 | Tricarboxylate transport protein, mitochondrial | -0.7 | | 0.01 |
| Ndufaf2 | NADH dehydrogenase [ubiquinone] 1 alpha subcomplex assembly factor 2 | -0.3 | | 0.01 |
| Mthfd1 | C-1-tetrahydrofolate synthase, cytoplasmic | 0.3 | | 0.01 |
| Rab6a | Ras-related protein Rab-6A | 0.3 | | 0.01 |
| Fmo1 | Dimethylaniline monooxygenase [N-oxide-forming] 1 | -0.3 | | 0.01 |
| Cavin2 | Caveolae-associated protein 2 | -0.3 | | 0.01 |
| Gba | Lysosomal acid glucosylceramidase | -0.3 | | 0.01 |
| Aacs | Acetoacetyl-CoA synthetase | -0.5 | | 0.01 |
| Dpep1 | Dipeptidase 1 | -0.3 | | 0.01 |
| Emilin2 | EMILIN-2 | -0.5 | | 0.01 |
| Me1 | NADP-dependent malic enzyme | -0.4 | | 0.01 |
| Col14a1 | Collagen alpha-1(XIV) chain | -0.3 | | 0.02 |
| Ywhag | 14-3-3 protein gamma | -0.3 | | 0.02 |
| Serpinh1 | Serpin H1 | -0.3 | | 0.02 |
| Gp1bb | Platelet glycoprotein Ib beta chain | 0.7 | | 0.02 |
| Prdx2 | Peroxiredoxin-2 | 0.5 | | 0.02 |
| Sptbn1 | Spectrin beta chain, non-erythrocytic 1 | -0.3 | | 0.02 |
| Thbs1 | Thrombospondin-1 | 0.6 | | 0.02 |
| Echdc1 | Ethylmalonyl-CoA decarboxylase | -0.4 | | 0.02 |
| Palmd | Palmdelphin | -0.4 | | 0.02 |
| Gpld1 | Phosphatidylinositol-glycan-specific phospholipase D | 0.6 | | 0.02 |
| Snx15 | Sorting nexin-15 | 0.3 | | 0.02 |
| Hagh | Hydroxyacylglutathione hydrolase, mitochondrial | 0.3 | | 0.02 |
| Rcc1 | Regulator of chromosome condensation | -0.3 | | 0.02 |
| Ig kappa chain V-III region PC 2880/PC 1229 | | 0.9 | | 0.02 |
| Col6a6 | Collagen alpha-6(VI) chain | -0.3 | | 0.02 |
| Alox12 | Polyunsaturated fatty acid lipoxygenase ALOX12 | 0.7 | | 0.02 |
| Steap4 | Metalloreductase STEAP4 | -0.3 | | 0.02 |
| Gp9 | Platelet glycoprotein IX | 0.8 | | 0.02 |
| Dhdh | Trans-1,2-dihydrobenzene-1,2-diol dehydrogenase | -0.5 | | 0.02 |
| Igh-3 | Ig gamma-2B chain C region | 0.7 | | 0.02 |
| Hint2 | Histidine triad nucleotide-binding protein 2, mitochondrial | -0.5 | | 0.02 |
| Por | NADPH--cytochrome P450 reductase | -0.3 | | 0.02 |
| Serpina6 | Corticosteroid-binding globulin | 0.6 | | 0.02 |
| Apeh | Acylamino-acid-releasing enzyme | 0.4 | | 0.02 |
| Agpat2 | 1-acyl-sn-glycerol-3-phosphate acyltransferase beta | -0.8 | | 0.02 |
| Slc16a10 | Monocarboxylate transporter 10 | 0.5 | | 0.02 |
| Folr2 | Folate receptor beta | -0.3 | | 0.02 |
| Mpp1 | 55 kDa erythrocyte membrane protein | 0.5 | | 0.02 |
| Sost | Sclerostin | 0.3 | | 0.02 |
| Pgm2l1 | Glucose 1,6-bisphosphate synthase | 0.4 | | 0.02 |
| Prkab1 | 5'-AMP-activated protein kinase subunit beta-1 | -0.3 | | 0.03 |
| Tpp2 | Tripeptidyl-peptidase 2 | 0.3 | | 0.03 |
| Dhrs7 | Dehydrogenase/reductase SDR family member 7 | -0.3 | | 0.03 |
| Slfn5 | Schlafen family member 5 | -0.3 | | 0.03 |
| Hsd17b4 | Peroxisomal multifunctional enzyme type 2 | -0.3 | | 0.03 |
| Snrpb | Small nuclear ribonucleoprotein-associated protein B | -0.3 | | 0.03 |
| Akap12 | A-kinase anchor protein 12 | -0.3 | | 0.03 |
| Ly6c1 | Lymphocyte antigen 6C1 | -0.4 | | 0.03 |
| S100a10 | Protein S100-A10 | -0.4 | | 0.03 |
| Clpp | ATP-dependent Clp protease proteolytic subunit, mitochondrial | -0.3 | | 0.03 |
| Aspa | Aspartoacylase | -0.6 | | 0.03 |
| Arf6 | ADP-ribosylation factor 6 | 0.3 | | 0.03 |
| Rpl18 | 60S ribosomal protein L18 | -0.4 | | 0.03 |
| Cma1 | Chymase | -0.6 | | 0.03 |
| Cnp | 2',3'-cyclic-nucleotide 3'-phosphodiesterase | 0.3 | | 0.03 |
| Tecr | Very-long-chain enoyl-CoA reductase | -0.4 | | 0.03 |
| Tuba4a | Tubulin alpha-4A chain | 0.3 | | 0.03 |
| Dbi | Acyl-CoA-binding protein | -0.5 | | 0.03 |
| Ube2o | (E3-independent) E2 ubiquitin-conjugating enzyme UBE2O | 0.3 | | 0.03 |
| Scrn3 | Secernin-3 | 0.3 | | 0.03 |
| Cisd1 | CDGSH iron-sulfur domain-containing protein 1 | -0.5 | | 0.03 |
| Trap1 | Heat shock protein 75 kDa, mitochondrial | -0.6 | | 0.03 |
| Acaa1a | 3-ketoacyl-CoA thiolase A, peroxisomal | -0.3 | | 0.03 |
| Sgpl1 | Sphingosine-1-phosphate lyase 1 | -0.4 | | 0.03 |
| Lcat | Phosphatidylcholine-sterol acyltransferase | 0.5 | | 0.03 |
| Ca1 | Carbonic anhydrase 1 | 0.6 | | 0.03 |
| Cyb5r1 | NADH-cytochrome b5 reductase 1 | -0.3 | | 0.03 |
| Hk2 | Hexokinase-2 | -0.5 | | 0.04 |
| Ig kappa chain V-V region HP 91A3 | | 0.8 | | 0.04 |
| Dock6 | Dedicator of cytokinesis protein 6 | -0.3 | | 0.04 |
| Exosc9 | Exosome complex component RRP45 | -0.3 | | 0.04 |
| Pvalb | Parvalbumin alpha | -1.3 | | 0.04 |
| Lyve1 | Lymphatic vessel endothelial hyaluronic acid receptor 1 | -0.3 | | 0.04 |
| Apoa4 | Apolipoprotein A-IV | 0.5 | | 0.04 |
| Tpm4 | Tropomyosin alpha-4 chain | 0.3 | | 0.04 |
| Lrrfip2 | Leucine-rich repeat flightless-interacting protein 2 | -0.4 | | 0.04 |
| Itga2b | Integrin alpha-IIb | 0.6 | | 0.04 |
| Slc25a10 | Mitochondrial dicarboxylate carrier | -0.5 | | 0.04 |
| Ube2l6 | Ubiquitin/ISG15-conjugating enzyme E2 L6 | 0.4 | | 0.04 |
| Coq7 | 5-demethoxyubiquinone hydroxylase, mitochondrial | -0.5 | | 0.04 |
| Rbm39 | RNA-binding protein 39 | -0.3 | | 0.04 |
| Mnda | Interferon-activable protein 205-B | -0.3 | | 0.04 |
| Cryzl1 | Quinone oxidoreductase-like protein 1 | -0.8 | | 0.04 |
| Aldh1l1 | Cytosolic 10-formyltetrahydrofolate dehydrogenase | -0.3 | | 0.04 |
| Chp1 | Calcineurin B homologous protein 1 | -0.3 | | 0.04 |
| Ephx2 | Bifunctional epoxide hydrolase 2 | -0.4 | | 0.04 |
| Mgll | Monoglyceride lipase | -0.5 | | 0.04 |
| Hsd17b12 | Very-long-chain 3-oxoacyl-CoA reductase | -0.3 | | 0.04 |
| Ig kappa chain V-III region ABPC 22/PC 9245 | | 1.0 | | 0.04 |
| Bid | BH3-interacting domain death agonist | 0.3 | | 0.04 |
| Mia2 | Melanoma inhibitory activity protein 2 | -0.3 | | 0.04 |
| Abcd3 | ATP-binding cassette sub-family D member 3 | -0.3 | | 0.04 |
| Clns1a | Methylosome subunit pICln | 0.4 | | 0.04 |
| Golt1b | Vesicle transport protein GOT1B | -0.3 | | 0.04 |
| Cyb5b | Cytochrome b5 type B | -0.3 | | 0.04 |
| Cd5l | CD5 antigen-like | 0.5 | | 0.04 |
| Rasgrp2 | RAS guanyl-releasing protein 2 | 0.3 | | 0.04 |
| Casp6 | Caspase-6 | 0.3 | | 0.04 |
| Lpin1 | Phosphatidate phosphatase LPIN1 | -0.5 | | 0.04 |
| Ighg1 | Ig gamma-1 chain C region secreted form | 0.6 | | 0.04 |
| Pygl | Glycogen phosphorylase, liver form | -0.4 | | 0.04 |
| Nudt9 | ADP-ribose pyrophosphatase, mitochondrial | 0.3 | | 0.04 |
| Hdhd3 | Haloacid dehalogenase-like hydrolase domain-containing protein 3 | -0.4 | | 0.04 |
| Stum | Protein stum homolog | 0.6 | | 0.04 |
| Tubb1 | Tubulin beta-1 chain | 0.6 | | 0.04 |
| Gapvd1 | GTPase-activating protein and VPS9 domain-containing protein 1 | 0.3 | | 0.04 |
| Usp15 | Ubiquitin carboxyl-terminal hydrolase 15 | 0.3 | | 0.04 |
| Ig heavy chain V region 6.96 | | 1.2 | | 0.04 |
| Aspn | Asporin | -0.3 | | 0.04 |
| Hba | Hemoglobin subunit alpha | 0.5 | | 0.05 |
| Pc | Pyruvate carboxylase, mitochondrial | -0.9 | | 0.05 |
| Slc4a1 | Band 3 anion transport protein | 0.6 | | 0.05 |
| Sptb | Spectrin beta chain, erythrocytic | 0.5 | | 0.05 |
| F5 | Coagulation factor V | 0.4 | | 0.05 |
| Macrod1 | ADP-ribose glycohydrolase MACROD1 | -0.4 | | 0.05 |
| Dmtn | Dematin | 0.4 | | 0.05 |
| Krt7 | Keratin, type II cytoskeletal 7 | -1.1 | | 0.05 |
| Mcpt4 | Mast cell protease 4 | -0.6 | | 0.05 |
| Ctnnbl1 | Beta-catenin-like protein 1 | -0.4 | | 0.05 |
| Stard10 | START domain-containing protein 10 | 0.4 | | 0.05 |
| Atp1b1 | Sodium/potassium-transporting ATPase subunit beta-1 | -0.3 | | 0.05 |
| Cenpv | Centromere protein V | -0.4 | | 0.05 |
| Rmdn3 | Regulator of microtubule dynamics protein 3 | -0.3 | | 0.05 |
| Snapin | SNARE-associated protein Snapin | -0.3 | | 0.05 |
| Rab8b | Ras-related protein Rab-8B | 0.3 | | 0.05 |
| Ig heavy chain V region 23 | | 0.7 | | 0.05 |
| Fabp4 | Fatty acid-binding protein, adipocyte | -0.9 | | 0.05 |
| Oxsm | 3-oxoacyl-[acyl-carrier-protein] synthase, mitochondrial | -0.3 | | 0.05 |
| Bpgm | Bisphosphoglycerate mutase | 0.5 | | 0.05 |
| Gclc | Glutamate--cysteine ligase catalytic subunit | 0.4 | | 0.05 |
| Rala | Ras-related protein Ral-A | -0.3 | | 0.05 |
| H1-2 | Histone H1.2 | -1.0 | | 0.05 |
| Smarca2 | Probable global transcription activator SNF2L2 | -0.3 | | 0.05 |
| Epb41 | Protein 4.1 | 0.5 | | 0.05 |
| Tmem205 | Transmembrane protein 205 | -0.4 | | 0.05 |
| Zadh2 | Prostaglandin reductase-3 | -0.3 | | 0.05 |
| C1qbp | Complement component 1 Q subcomponent-binding protein, mitochondrial | -0.3 | | 0.05 |
| Tfrc | Transferrin receptor protein 1 | 0.3 | | 0.05 |
| Spta1 | Spectrin alpha chain, erythrocytic 1 | 0.5 | | 0.05 |
| Ckm | Creatine kinase M-type | -1.1 | | 0.05 |
| Ig alpha chain C region | | 0.7 | | 0.05 |
| Prdx5 | Peroxiredoxin-5, mitochondrial | -0.3 | | 0.05 |
| Lipe | Hormone-sensitive lipase | -0.8 | | 0.05 |
| Add2 | Beta-adducin | 0.4 | | 0.05 |
| Stx2 | Syntaxin-2 | -0.3 | | 0.05 |
| Eif5 | Eukaryotic translation initiation factor 5 | 0.3 | | 0.05 |
| Cpa3 | Mast cell carboxypeptidase A | -0.6 | | 0.05 |
| Aldh4a1 | Delta-1-pyrroline-5-carboxylate dehydrogenase, mitochondrial | -0.3 | | 0.05 |
| Srp14 | Signal recognition particle 14 kDa protein | -0.4 | | 0.05 |
| Dcn | Decorin | -0.3 | | 0.05 |
| Dolk | Dolichol kinase | -0.3 | | 0.05 |
| H2-Q10 | H-2 class I histocompatibility antigen, Q10 alpha chain | 0.4 | | 0.05 |
| Sec14l2 | SEC14-like protein 2 | 0.4 | | 0.05 |
| Acsf2 | Medium-chain acyl-CoA ligase ACSF2, mitochondrial | -0.3 | | 0.05 |
| Rpl27 | 60S ribosomal protein L27 | -0.3 | | 0.05 |
